# Role of Pro-Inflammatory Cytokines and Vitamin D in Probable Alzheimer's Disease with Depression

**DOI:** 10.14336/AD.2016.1017

**Published:** 2017-05-02

**Authors:** Anindita Banerjee, Vineet Kumar Khemka, Debashree Roy, Aparajita Dhar, Tapan Kumar Sinha Roy, Atanu Biswas, Barun Mukhopadhyay, Sasanka Chakrabarti

**Affiliations:** ^1^Department of Biochemistry, ICARE Institute of Medical Sciences and Research, Haldia, India.; ^2^Department of Biochemistry, Institute of Post Graduate Medical Education and Research, Kolkata, India.; ^3^Department of Neurology, Bangur Institute of Neurosciences, Kolkata, India.; ^4^Biological Anthropology Unit, Indian Statistical Institute, Kolkata, India.

**Keywords:** IL 6, TNF α, 25-Hydroxyvitamin D, Neurodegeneration, Amyloid beta peptide

## Abstract

Symptoms of depression are present in a significant proportion of Alzheimer's disease (AD) patients. While epidemiological studies have shown a strong association between depression and AD, it has not been established whether depression is a risk factor or merely a co-morbidity of AD. It is also uncertain if depression affects the pathogenesis of AD. In this paper, we address these questions by measuring the serum levels of two common metabolic risk factors of AD and depression, inflammatory cytokines (IL 6 and TNF alpha) and serum 25-hydroxyvitamin D, in a case-control study. We measured the serum levels of IL 6, TNF α and 25-hydroxyvitamin D in age-matched healthy controls (n= 60) and in AD patients without depression (n=26) or AD patients with depression (n=34), and statistically analyzed the changes in these parameters among different groups under this study. Our results show that in AD there is a significant increase in IL 6 and TNF α and a marked decrease in 25-hydroxyvitamin D in the peripheral circulation compared to age-matched healthy controls. Furthermore, AD patients with depression have even significantly higher levels of IL 6 or TNF α and a lower level of 25-hydroxyvitamin D in circulation than in AD patients without depression. We also found a strong statistical correlation between the disease severity and the serum levels of IL 6, TNF α and 25-hydroxyvitamin D in AD patients with depression. These results suggest that altered circulating levels of common metabolic risk factors lead to the co-existence of depression with AD in many patients, and when they co-exist, the depression presumably affects the severity of AD presentations through more aggravated changes in these risk factors.

Alzheimer’s disease (AD) is the most common neurodegenerative disease affecting the elderly population leading to progressive loss of memory and multiple cognitive functions [1]. Nearly 95% of AD patients suffer from the sporadic form of the disease, while the rest with identifiable mutations in APP (amyloid precursor protein) or PS1 (Presenilin 1) or PS2 (Presenilin 2) gene belong to the familial variety of AD [2,3]. More than two decades of experimental research and post-mortem studies have identified several pathogenic mechanisms in the AD brain such as the accumulation of amyloid beta peptide (predominantly Aβ 42 and Aβ 40) and phosphorylated tau protein, oxidative damage to specific proteins or in general to lipid, protein and DNA, mitochondrial dysfunction and inflammatory response [4-11]. It is plausible that in the familial form these pathogenic mechanisms are triggered by gene mutations, while in the sporadic variety the pathogenesis is the result of an interplay of multiple environmental risk factors, gene polymorphisms and aging [3,12-15].

Apart from dementia and cognitive decline, various behavioral and psychological features are also present in AD having physical, emotional and financial impact on the patients [16]. Depression is one of the most common behavioral and psychological symptoms present in AD patients leading to a more rapid clinical deterioration [16]. Several cross-sectional and longitudinal studies have found an association between late-life depression and subsequent cognitive decline to MCI (mild cognitive impairment) and dementia [17-19]. A recent systematic review and meta-analysis found a two-fold increase in risk of dementia in depressed patients [20], and it has been suggested that a significant number of AD cases worldwide could be attributed to depression [20]. Depressive symptoms, therefore, may reflect an underlying neuropathologic condition that manifests as cognitive decline over time, but equally depression could be an independent risk factor for AD. It is interesting to note that several established metabolic risk factors of AD have also clear links with depression as evident from separate epidemiological studies. For example, epidemiological case-control and large population based follow-up studies have indicated the association of low circulating levels of 25-hydroxyvitamin D with cognitive decline and Alzheimer’s disease [21-24]. On the other hand, several epidemiological studies have also observed that the low serum 25-hydroxyvitamin D level is associated with depression [25,26]. With respect to pro-inflammatory cytokines, epidemiological studies have shown the association of elevated levels of IL 1, IL 6, TNF α and others in peripheral circulation with AD [27-29]. Additionally, different studies have also reported increased circulating levels of pro-inflammatory cytokines like IL 1, IL 6 and TNF α in major depression patients [30,31].

Apart from epidemiological studies, the experimental research has indicated the molecular mechanisms through which circulating levels of vitamin D could affect neuronal plasticity, neurogenesis, neurotransmitter turnover, and other brain functions that have obvious implications in mood, behavior, memory and cognition [32,33]. Likewise, it has been established that elevated circulating pro-inflammatory cytokines through several well-defined mechanisms can increase the CNS cytokines which in turn can impact the mood, memory and cognition through alterations of synaptic properties, neurogenesis and neurotransmission at different brain regions as well as by changes in the amyloid beta peptide metabolism [34,35]. Thus, it is likely from the collective evidence from experimental and epidemiological studies that a complex relationship exists among AD and depression through the operations of risk factors like the lower level of vitamin D and elevated levels of pro-inflammatory cytokines in peripheral circulation. In this context it is worthwhile to measure these serum parameters simultaneously in controls and AD patients with and without depression and to examine the statistical correlations among different variables.

## MATERIALS AND METHODS

### Subjects

The study included 60 AD patients and 60 control subjects. The patients for this study were selected from the ‘dementia clinic’ of Bangur Institute of Neurosciences, Kolkata, which is a tertiary care hospital in the eastern part of India. The healthy control subjects were age and sex matched volunteers chosen randomly from the various free health camps organized by us for the general population. Informed consent was obtained from every control subject and in the case of AD patients from a near relative accompanying the patient. The study was cleared by the Institutional Human Ethics Committee according to Helsinki guidelines. The diagnosis of probable AD was made by neuropsychological evaluation as per DSM - IV (Diagnostic and Statistical Manual of Mental Disorders) criteria supported by MRI findings. Neuropsychiatric evaluation and disease severity assessment included the determination of MMSE scores (out of 30) through a series of questions and tests for assessing the patient's memory, attention, orientation, registration and reasoning. A maximum score of 30 was attainable by a person without any neuropsychological impairment, while AD cases had decreased MMSE scores reflecting the severity of the disease. The patients were further divided into two sub groups: AD without depression (n = 26) and AD with depression (n = 34) diagnosed as per DSM-IV diagnostic criteria for major depressive disorder and depressive episodes. All the healthy control subjects were also examined clinically to exclude any cognitive impairment. The exclusion criteria in AD group included diabetes mellitus, overt cardiovascular disease, chronic kidney disease, cancer, chronic infection, and any other associated neurological disease.

### Biochemical Assays

Non-fasting serum samples of control and AD subjects were analyzed for routine biochemical parameters immediately after collection while aliquots of the samples were also stored at - 20° C for the assay of 25-hydroxyvitamin D, IL 6 and TNF α.

**Table 1:** Demographic and biochemical profile of the subjects

	Control (n = 60)	AD with depression (n = 34)	AD without depression (n = 26)
Age (in years)	71.95 ± 6.27	70.20 ± 5.17	69.28 ± 4.67
Sex (M/F)	31/29	18/16	14/12
BMI (kg/m^2^)	22.03 ± 1.39	21.66 ± 1.00	22.42± 0.88
Glucose (mg/dl)	88.13 ± 11.64	89.10 ± 11.36	84.22 ± 12.48
Cholesterol (mg/dl)	174.70 ± 16.49	213.30 ± 23.17 ^*^	203.8 ± 28.32^*^

### Immunoassays

Serum vitamin D was measured as 25-hydroxyvitamin D considered as the indicator of vitamin D reserve in the body, and thus these two terms are used interchangeably in this manuscript. 25-hydroxyvitamin D was measured by using commercially available ELISA kits (Calbiotech, USA). Briefly, anti-25-hydroxyvitamin D antibody (capture antibody) coated wells were incubated with standards (25-hydroxyvitamin D), samples and vitamin D-biotin conjugate at room temperature for 90 minutes. The binding of vitamin D-biotin conjugate to the wells by the capture antibody decreased by competition with 25-hydroxyvitamin D present in the standards or samples. Following a wash step, bound vitamin D-biotin was detected with streptavidin-horse radish peroxidase (SA-HRP) using tetramethylbenzidine (TMB) as the substrate. For drawing the calibration curve from the measured absorbance readings, a 4- parametric logistic (4-PL) curve was used. Serum IL 6 and TNF α were assayed by solid-phase sandwich ELISA using commercial kits from Raybiotech, USA and Invitrogen, USA respectively following the manufacturer’s protocol. For IL 6 or TNF α assay, anti-human IL 6 antibody or anti-human TNF α antibody coated microtiter plates were used to capture IL 6 or TNF α respectively from the standards or the samples (diluted as per the manufacturer's instructions). The enzyme-conjugated detector antibody was then added to bind to the target (IL 6 or TNF α) to form the sandwich. Following incubation and washing, the substrate (TMB) was added to develop the color. The calibration curves were drawn using standards from 0 - 1000 pg/ml (TNF α) or 1.37 - 1000 pg / ml (IL 6) respectively.

### Statistical analysis

For two normally distributed sample groups, the means were compared by Student’s unpaired ‘t’ test. To find out the correlation between two variables, Pearson’s product moment correlation coefficient or Spearman nonparametric correlation coefficient was used. A value of p < 0.05 was considered as statistically significant. The direct or inverse correlation was indicated by a positive or negative ‘r’ value respectively. The statistical analysis was performed by using Graph Pad prism software (version 5, 2007, Sandiego, California, USA).

**Figure 1.**
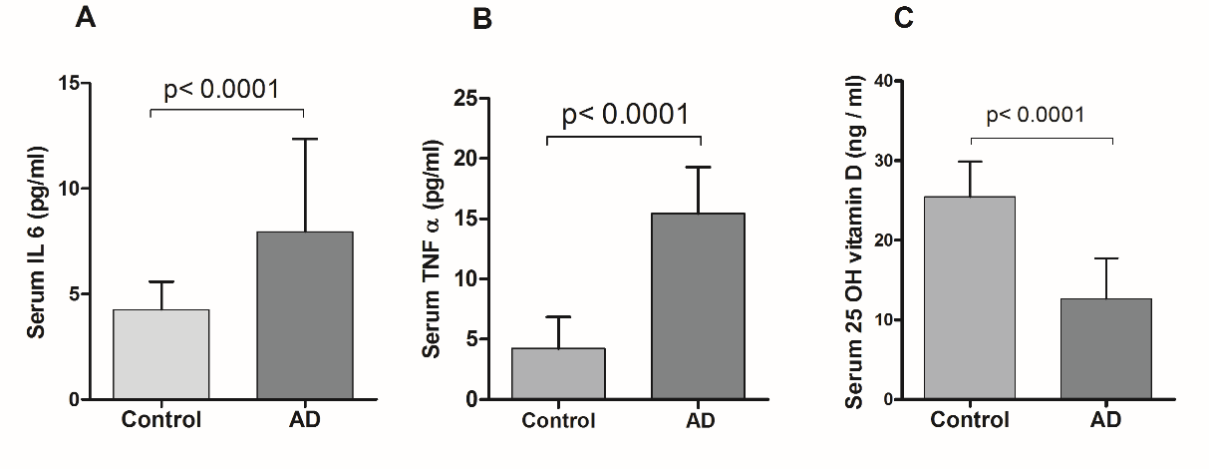
Serum levels of IL 6, TNF α and 25-hydroxyvitamin D (25 OH vitamin D) in AD subjects Values are expressed as the means ± SD for the number of cases in each group of subjects. Statistically significant, *p*<0.0001 AD vs. Control.

**Figure 2.**
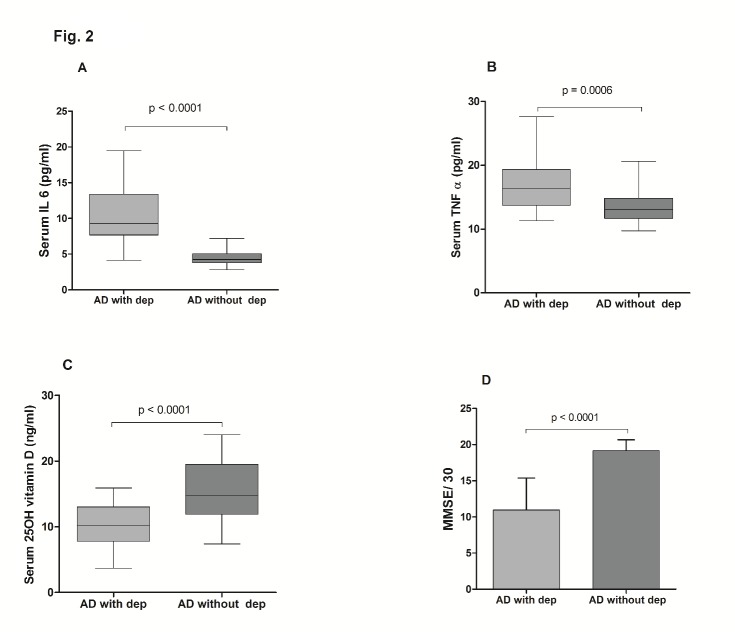
Serum levels of IL 6, TNF α, 25-hydroxyvitamin D (25OH vitamin D) and MMSE in AD subjects with depression (AD with dep) and without depression (AD without dep) Box and Whisker plots represent median, upper median, lower median, and minimum to maximum range of serum IL 6 **(A)**, TNF α **(B)** and 25-hydroxyvitamin D **(C)** while Bar diagram represents mean MMSE **(D)** among AD subjects with and without depression. Values expressed as the means ± SD are: IL 6 (pg/ml) 10.47±4.18 (AD with depression), 4.56±1.20 (AD without depression); TNFα (pg/ml) 16.84±3.88 (AD with depression), 13.48±2.92 (AD without depression); 25-hydroxyvitamin D (ng/ml) 10.22 ± 3.22 (AD with depression), 15.61 ± 4.64 (AD without depression); MMSE 10.9±4.38 (AD with depression), 19.15±1.46 (AD without depression). Statistically significant difference, p < 0.001, AD subjects with depression versus AD subjects without depression.

## RESULTS

**Figure 3.**
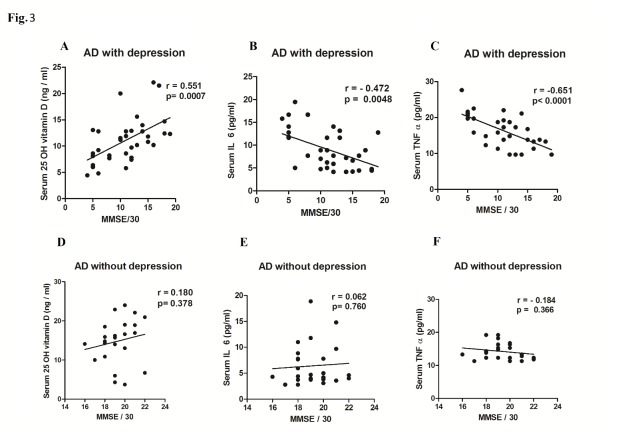
Correlation between MMSE scores and serum levels of 25-hydroxyviamin D (25 OH vitamin D) or IL 6 orTNF α in AD with or without depression. XY scatter plots are shown between MMSE scores and serum levels of 25-hydroxyvitamin D (A) or IL 6 (B) or TNF α (C) in AD subjects with depression or serum levels of 25-hydroxyvitamin D (D) or IL 6 (E) or TNF α (F) in AD subjects without depression. The degree and nature of correlation between the MMSE score and the serum parameter in AD patients is given by the value of r (correlation coefficient) as explained in the methods. A value of *p* < 0.05 was considered as statistically significant.

**Figure 4.**
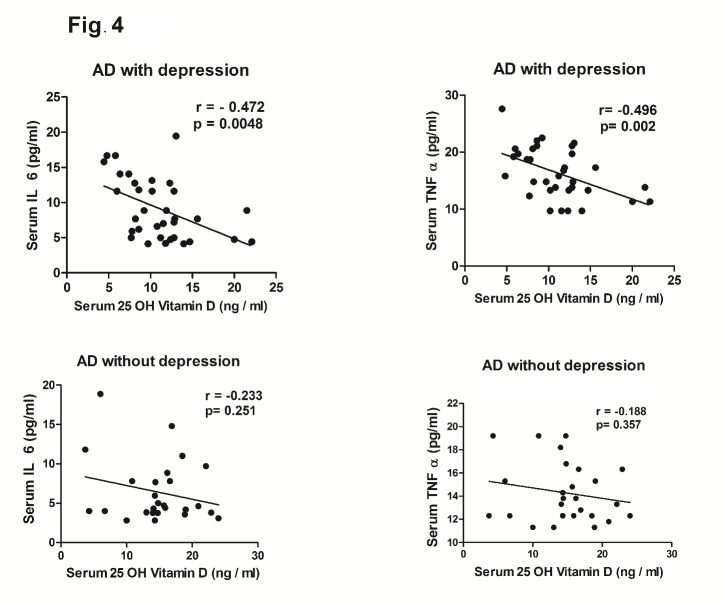
XY scatter plots between serum levels of 25-hydroxyvitamin D (25 OH Vitamin D) and IL 6 or TNF α in AD with or without depression. The degree and nature of correlation between the MMSE score and the serum parameter in AD patients is given by the value of r (correlation coefficient) as explained in the methods. A value of *p* < 0.05 was considered as statistically significant.

The demographic profile of the subjects (Table 1) showed no statistically significant difference in age, sex distribution and BMI among the three groups. Routine biochemical parameters were within normal limits for all the groups (Table 1) and in accordance with our exclusion criteria for AD patients (complete data set not presented) except for mildly elevated serum total cholesterol in both the AD subgroups as compared to healthy controls (*p*<0.001). The serum IL 6 (Mean ± SD) was conspicuously higher in AD subjects (7.95 ± 4.39 pg/ml) as compared to healthy controls (4.2 ± 1.3 pg/ml) and the difference was statistically significant (*p*<0.0001) as shown in Fig. 1A. Moreover, the serum TNF α level was also more than 3.5-fold higher in AD subjects than in healthy controls (*p*<0.0001) as shown in Fig. 1B. On the other hand, serum 25-hydroxyvitamin D was lower by nearly 50% in AD subjects with respect to controls (Fig. 1C). When these parameters were compared between two sub groups of AD subjects, it was observed that serum IL 6 and TNF α levels were significantly higher (*p*<0.001 and *p*<0.0006 respectively) in depressive AD patients than their counterparts without depression (Fig. 2A, 2B). Further, as shown in Fig. 2C, serum 25-hydroxyvitamin D was significantly (*p*<0.0001) lower in AD subjects with depression (10.22 ± 3.22 ng/ml) than in AD without depression (15.61 ± 4.64 ng/ml). Further, the mean MMSE score of depressive AD patients was significantly (*p*<0.0001) lower (10.9 ± 4.38) than their non-depressed counterparts (19.15 ± 1.46) as shown in Fig. 2D. A strong direct correlation was observed between MMSE and serum 25-hydroxyvitamin D in AD subjects with depression (r = 0.55, p = 0.007) but not in AD patients without depression (Fig. 3A, 3D). In AD with depression group, a moderate inverse correlation was seen between serum IL 6 levels and MMSE score (r = - 0.47, *p* = 0.0048), while a strong inverse correlation existed between serum TNF α levels and MMSE scores (r = - 0.65, p = 0.0001) (Fig. 3B, 3C). In AD patients without depression, the weak correlation observed between MMSE and IL 6 or TNF α were not statistically significant (Fig. 3E, 3F). In control subjects also no correlation was found between the MMSE scores and any of the above parameters (data not shown). Moreover, a significant inverse correlation was observed between serum levels of 25-hydroxyvitamin D and IL 6 (r = - 0.47, *p* = 0.0048) or TNF α (r = - 0.49, *p* = 0.002) in AD subjects with depression, but not in AD without depression (Fig. 4).

## DISCUSSION

Although epidemiological studies have strongly indicated the association of depression with AD, it is not firmly established whether depression is a risk factor for AD, a co-morbidity or the manifestation of an underlying neurodegeneration [17,36]. Likewise, there are conflicting reports of the role of depression in the progression from MCI to clinical AD as evidenced in several longitudinal studies [37,38]. Furthermore, there is no systematic study to indicate that depression when present in AD subjects substantially alters the clinical progression or severity of AD. The post-mortem brains of patients with major depression exhibit morphological changes with decreased number of neurons, presumably because of reduced neurogenesis, but frank neurodegeneration or AD-like pathology is not apparent in such cases [39-41]. On the other hand, shared molecular and cellular mechanisms are present in the pathogenesis of AD and major depression [39,42]. Further, depression is not a feature of all AD patients, but present in approximately 25 - 40% of cases [43]. Thus the relationship of AD and depression appears to be complex and needs further explorations. We envisage that common risk factors involved in the pathogenesis of AD and depression would explain the co-existence of both these conditions in the same patients. In this study we have not attempted to identify risk factors for AD and depression, but instead have chosen circulating IL 6, TNF α and 25-hydroxyvitamin D as our study parameters because of the accumulated evidence that elevated pro-inflammatory cytokines or decreased 25-hydroxyvitamin D in peripheral circulation are causally associated with and potential risk factors for AD and depression [21,26,29,31].

In an earlier study we have observed that AD patients with associated depression have higher circulating levels of pro-inflammatory cytokines than those without depression [44]. In the present study, we have further extended our observation by measuring both pro-inflammatory cytokines and 25-hydroxyvitamin D in circulation in AD patients with or without depression. Firstly, the results of this cross-sectional study (Fig. 1) are in conformity with the currently held view that increased IL 6 and TNF α and decreased 25-hydroxyvitamin D in peripheral circulation are potential risk factors for AD [21,29]. In addition, the results are more revealing when these parameters are compared between two subgroups of AD patients with and without depression. Our data suggests that marked alterations in the circulating levels of IL 6, TNF α and 25-hydroxyvitamin D (Fig. 2) result in severe AD with low MMSE and depression as the co-morbidity, while less dramatic changes of these parameters lead to AD with moderately low MMSE but without depression (Fig. 2). Further in AD with depression group, the serum levels of IL 6, TNF α and 25-hydroxyvitamin D show a strong correlation with MMSE scores which implies that marked alterations of these parameters have a causal role in the progression of AD pathogenesis (Fig. 3). This is, however, not the case when the levels of these risk factors are only moderately altered as in AD without depression (Fig. 3). Although the raised levels of circulating pro-inflammatory cytokines have been shown to be associated with AD in many studies, the relationship of these alterations with MMSE scores or other indices of disease severity has not been established firmly [27,45,46]. Our results do indicate a causal association of such alterations in pro-inflammatory cytokines or vitamin D with disease progression which is in agreement with our earlier published study [44]. Another study has reported that serum level of TNF α is lower in mild or moderate AD than in the severe form of the disease which tends to support our data [47].

Recently, inflammatory response in the brain has been held as the common mechanism of depression and neurodegeneration through varied actions of pro-inflammatory cytokines [34,35,39,48,49]. Although the exact reasons for the rise of proinflammatory cytokines in AD or depression are not known, the pathways by which peripheral cytokines augment the levels of CNS cytokines are well established [35,50]. The suggested pathways include the activation of peripheral vagal afferents, formation of diffusible messengers in the cerebral blood vessels and direct entry into the brain through a region of deficient BBB such as the Organum Vasculosum of Lamina Terminalis [35,50-52]. In turn, the raised levels of CNS cytokines can cause alterations of synaptic properties, neurigenesis, neurotransmission and the activity of the hypothalamic - pituitary -adrenal axis which may all contribute to the genesis of AD and depression [35,50-52]. Apart from the proinflammatory cytokines, a lower level of vitamin D could also be causal to the pathogenesis of both AD and depression especially because vitamin D affects neuronal plasticity and the brain immune response, facilitates amyloid beta clearance from the brain and exhibits neurotrophic, neuroprotective and antioxidant effects in different experimental conditions [53-55]. Another interesting and significant correlation is seen between the levels of vitamin D and IL 6 or TNF α in the AD with depression group, but not in controls or AD without depression (Fig. 4). Vitamin D has immunomodulatory activity and can inhibit the production of proinflammatory cytokines like IL 6 or TNF α in monocytes and macrophages via the inhibition of p38 MAP kinase pathway and VDR mediated interaction and inhibition of NF-κB signaling [56,57]. Thus the significantly low level of serum vitamin D in AD patients with depression may have contributed to the raised level of circulating IL 6 and TNF α in such patients.

There are, however, some confounding factors that may have affected our results. The demographic profile of three groups of subjects does not indicate any significant difference among these groups in terms of age, gender, BMI or common serum biochemical parameters (Table 1), but AD patients were under different types of drugs and dosage schedules. Moreover, AD patients with depression were advised anti-depressants belonging to SSRI (selective serotonin reuptake inhibitor) and tricyclic antidepressants. All these might have confounded our results to an extent, but nevertheless the alterations reported in the present study are very convincingly different in two subgroups of AD subjects and in age-matched controls.

Notwithstanding the limitations of a case-control study, the analysis of our data and the suggested underlying biochemical and pathophysiological mechanisms clearly indicate that common metabolic risk factors may lead to the co-existence of depression with AD. The moot point whether the co-existence of depression with AD alters the clinical severity or progression of the latter disorder may not be settled so easily. An elaborate follow-up study of MCI to AD with different degrees of severity in the presence or absence of depression along with the measurements of the relevant metabolic risk factors and brain amyloid imaging at different time points could be useful in this context. However, in the present study the AD subjects with depression have significantly lower values of MMSE scores and greater alterations of circulating proinflammatory cytokines and vitamin D, and this indicates but does not confirm that a co-existing depression affects the severity of AD through changes in such metabolic risk factors. Furthermore, our results strongly support the potential of therapeutic intervention, particularly in cases of AD with depression, by vitamin D supplementation or treatment with specific receptor antagonists of pro-inflammatory cytokines.
